# Declarative Learning Mechanisms Support Declarative but Not Probabilistic Feedback-Based Learning in Children with Developmental Language Disorder (DLD)

**DOI:** 10.3390/brainsci13121649

**Published:** 2023-11-28

**Authors:** Asiya Gul, Lauren S. Baron, Kelsey B. Black, Annika L. Schafer, Yael Arbel

**Affiliations:** MGH Institute of Health Professions, Boston, MA 02129, USA; agul@mghihp.edu (A.G.); lbaron@mghihp.edu (L.S.B.); kblack9@mgb.org (K.B.B.); alschafer@mgb.org (A.L.S.)

**Keywords:** developmental language disorder, feedback-based learning, declarative learning, probabilistic learning, feedback-related negativity, event-related potentials, N170

## Abstract

Declarative and probabilistic feedback-based learning was evaluated in 8–12-year-old school-age children with developmental language disorder (DLD; *n* = 14) and age-matched children with typical development (TD; *n* = 15). Children performed a visual two-choice word-learning task and a visual probabilistic classification task while their electroencephalogram (EEG) was recorded non-invasively from the scalp. Behavioral measures of accuracy and response to feedback, and electrophysiological responses to feedback were collected and compared between the two groups. While behavioral data indicated poorer performance by children with DLD in both learning paradigms, and similar response patterns to positive and negative feedback, electrophysiological data highlighted processing patterns in the DLD group that differed by task. More specifically, in this group, feedback processing in the context of declarative learning, which is known to be dominated by the medial temporal lobe (MTL), was associated with enhanced N170, an event-related brain potential (ERP) associated with MTL activation. The N170 amplitude was found to be correlated with declarative task performance in the DLD group. During probabilistic learning, known to be governed by the striatal-based learning system, the feedback-related negativity (FRN) ERP, which is the product of the cortico-striatal circuit dominated feedback processing. Within the context of probabilistic learning, enhanced N170 was associated with poor learning in the TD group, suggesting that MTL activation during probabilistic learning disrupts learning. These results are interpreted within the context of a proposed feedback parity hypothesis suggesting that in children with DLD, the system that dominates learning (i.e., MTL during declarative learning and the striatum during probabilistic learning) dominates and supports feedback processing.

## 1. Introduction

Feedback is an integral part of various teaching methods and intervention techniques used with children. For a learner, it is crucial to use the information carried by external feedback effectively, to guide learning. Recent findings suggest that children with developmental language disorder (DLD) exhibit atypical feedback processing, which may contribute to their difficulties in learning and language acquisition [1,2,3]. Notably, DLD is defined as a neurodevelopmental disorder that affects language development and that cannot be attributed to hearing loss or other known acquired and developmental neurological disorders [4]. Children with DLD also commonly demonstrate weaknesses in cognitive skills such as working memory [5,6,7,8], attention [9,10], and other executive functions [11,12,13]. DLD occurs in approximately 8% of school-aged children [14,15,16], and the potential impacts of DLD are pervasive and severe [17]. Academically, children with DLD are six times more likely to develop a reading disability and four times more likely to develop a math disability compared to peers with typical development (TD) [18]. Children with DLD also have an increased risk of social–emotional challenges and marked difficulties with peer relationships, which extend to adolescence [19,20]. Different brain systems have been implicated in the processing of feedback. Particularly relevant to the study of DLD is the contribution of the striatum and the medial temporal lobe (MTL) to feedback-based learning. The striatum supports reinforcement [21,22,23], while the medial temporal lobe contributes to declarative learning. The relevance of these brain circuits to DLD is rooted in an ongoing debate about the neurophysiological deficit that underlies the disorder. The Procedural Deficit Hypothesis (PDH) suggests that DLD is largely attributed to an abnormal procedural memory system, which contributes to implicit learning [24]. This complex network includes the frontal/striatal circuits that support motor, cognitive, and grammar learning. The PDH further suggests that cognitive processes reliant on the MTL declarative learning system are spared in children with DLD [25]. The question of whether children with DLD derive greater benefit from declarative or probabilistic learning mechanisms remains unsettled. This uncertainty arises from conflicting evidence. One body of research suggests that children with DLD exhibit poor implicit learning and possess a relatively stable declarative learning system [24]. On the other hand, there is ample evidence that children with DLD exhibit limitations in the cognitive resources that are critical for declarative learning (see [26]) and that they benefit when implicit learning principles are implemented during intervention [27,28,29,30,31,32,33]. Evidence of difficulties extracting statistical patterns from linguistic input [24,34,35] and reported abnormalities among individuals with DLD in the striatum and prefrontal cortex [36,37,38,39,40] strengthen the PDH hypothesis. On the other hand, there is evidence that intervention approaches rooted in striatum-based statistical learning facilitate learning in children with DLD [27,28,29,30,31,32,33]. Such methods include increased exemplar variability [27,28,29,30,31,33], auditory bombardment [32], and cross-situational learning [41].

The study of feedback-based learning can shed light on the contribution of the striatum and MTL to learning in DLD because both learning systems can be activated to facilitate feedback processing. The role of striatal dopaminergic neurons in feedback-based learning has been well established, particularly in the context of reinforcement learning [22,42,43,44,45]. Additionally, individuals with disorders associated with striatal dysfunction, such as Parkinson’s disease and Huntington’s disease, have demonstrated poor feedback-based learning [44,46]. The same individuals demonstrated intact learning when feedback was manipulated to activate their spared MTL learning system [23]. Evidence suggests that the striatum-based and MTL-based learning systems interact and compete during the learning process [47,48,49,50,51], with each system becoming dominant, based on learning demands. While striatum-based implicit learning dominates complex and incremental (gradual and based on the accumulation of implicit information) learning, MTL-based declarative learning is ideal for learning facts, simple rules, and associations that can be expressed verbally by the learner [52,53,54,55]. Damage to the basal ganglia, such as in Parkinson’s disease, impairs incremental feedback-based learning, or the ability to accrue information on the likelihood of actions to lead to rewarding outcomes [22,56]. Damage to the MTL found in people with amnesia impairs declarative learning [22,57,58,59]. Evidence suggests that damage to one system leads to the dominance of the intact system [50,60,61,62,63]. However, it is yet unclear if this dominance facilitates learning [50]. It is important to note that one should not assume that patterns observed in adults with acquired damage to learning systems will be similar to those found in the developing brains of children with language impairments.

### 1.1. The Feedback Learning Parity (FLP) Theory

While there is agreement that children with DLD demonstrate difficulties extracting statistical patterns from the input, there is an ongoing debate about the role of the declarative learning system as either a compensatory or a disrupting mechanism. The PDH suggests that the MTL declarative learning system serves as a compensatory mechanism. We propose the feedback learning parity (FLP) hypothesis, whereby declarative learning mechanisms may support MTL-based declarative learning but disrupt striatal-based implicit learning in children with DLD. This hypothesis is based on evidence that children with DLD rely heavily on declarative learning, particularly rote learning [64,65,66,67,68], even when such techniques are proven inefficient for complex learning, as they do not promote generalization. For example, Gopnik and Crago [64] suggested that children with DLD engage in exemplar-based learning of regular and irregular past-tense verbs. Others have reported similar tendencies by children with DLD to engage in rote learning, reflected in a narrower use of learned forms [65] and context-dependent use of newly acquired noun phrases [67]. Additionally, in children and adults without DLD, poor performance on complex learning tasks has been linked to extensive use of ineffective declarative strategies (e.g., relying on a single dimension/rule), see [49,69]). In Parkinson’s disease, which is characterized by damage to the striatum, there is consistent evidence of enhanced MTL activation, even during tasks that require implicit learning [56,62]. However, while some reports indicate that such activation is present within the context of intact task performance [61,62], in others, MTL activation is coupled with poor task performance [60,63]. The former reports suggest that the MTL may serve as an effective compensatory mechanism, while the latter implies that MTL activation does not lead to improved learning. Our proposed FLP hypothesis suggests that in children with DLD, MTL-based feedback processing is enhanced and beneficial in the context of MTL-based declarative learning, and striatum-based feedback processing supports striatum-based probabilistic learning. We suggest that learning difficulties in DLD may stem, at least in part, from impaired communication/cooperation between the two learning systems (MTL and striatum-based systems). The proposed FLP is based on evidence that cooperation between the MTL and striatum-based learning systems is mediated by the prefrontal cortex [70,71,72,73] and is critical for the generalization of information across contexts, a known weakness among individuals with DLD [64,67,74,75].

### 1.2. Electrophysiological Measures of Striatal-Based and MTL-Based Feedback Processing

Electrophysiological measures in the time and time–frequency domains are optimal measures of processing that the learner may not be aware of and are not captured by behavioral measures of reaction time or accuracy. The N170 and FRN event-related potentials (ERPs) recorded from the scalp during learning are thought to capture MTL and striatum involvement in feedback-based learning, respectively. The FRN is a marker of reinforcement learning generated by the cortico-striatal circuit. It has been proposed that the FRN is a product of reinforcement learning signals carried by the mesencephalic dopamine system (MDS) to the anterior cingulate cortex when the monitoring system in the basal ganglia evaluates the outcomes of internal and external events [22,76,77,78]. The FRN has been found to capture differences in feedback processing between individuals with intact and impaired cortico-striatal circuits [46,79], making it a reliable measure of striatum intactness. It has been studied extensively in probabilistic learning tasks that require incremental feedback-based learning [22,80,81] and in paired-associate declarative learning tasks with deterministic feedback on a trial-by-trial basis [1,82,83,84]. Previous studies have used time–frequency decomposition of the FRN and found two distinct low-frequency oscillatory signals that reflect different aspects of feedback processing. The theta (~4–7 Hz) signal is assumed to be generated by the ACC in response to negative feedback or losses, and is thought to be related to cognitive control and error correction. The delta (~1–3 Hz) signal is suggested to originate from the striatum in response to positive feedback or gains [85,86,87,88], to be sensitive to reward magnitude, and to reflect reward processing and learning [85,86,87,88,89,90,91]. These signals, which have different temporal and functional characteristics, contribute to the processing of positive and negative feedback, with the theta dominating the FRN elicited by negative feedback and the delta dominating the FRN signal elicited by positive feedback.

The N170, which is known to be elicited in visuospatial tasks [92,93,94] and reflects MTL activation [95], captures feedback processing that is governed by the MTL. More specifically, its amplitude is sensitive to manipulations of feedback timing known to engage or disengage the MTL in the learning process [82,96,97]. Within the context of feedback processing, the MTL serves to create associations in long-term memory between responses and outcomes, while the striatum engages in reinforcement learning.

### 1.3. Current Study

The present study examines feedback processing in children with DLD in the context of declarative and probabilistic learning. It aims to evaluate (1) the striatum-based processing as captured by the FRN, (2) the MTL-based processing as captured by the N170 in the two learning paradigms, and (3) their association with learning outcomes. Although both tasks are feedback-based, they differ in the type of processing needed to extract information from the feedback to support learning. In declarative learning tasks, the feedback is deterministic, such that it provides the learner with information that can be used on a trial-by-trial basis to facilitate performance and guide paired-associate learning. In a two-choice paired-associate declarative learning task, positive feedback solidifies hypothesized associations, while negative feedback indicates the need to switch a response. This type of learning is thought to be supported by the MTL. In probabilistic learning paradigms, on the other hand, the information provided by the feedback is incremental, and learning from feedback requires the gradual accumulation of knowledge over many trials [59,98]. Probabilistic feedback-based learning relies on the intactness of basal ganglia, particularly the striatum [99,100,101]. The examination of the FRN and N170 ERP components, their lower frequency composition, and their association with declarative and probabilistic learning outcomes will shed light on the role of the MTL and striatum in feedback-based learning in children with DLD.

### 1.4. Hypotheses

The hypothesis of the procedural deficit theory, that the MTL serves as a compensatory mechanism in children with DLD, would be supported by evidence of greater MTL activation (N170) in this group in both learning paradigms, with a positive association between this activation and learning outcomes. The feedback learning parity (FLP) hypothesis suggests that in children with DLD’s reliance on declarative learning mechanisms supports declarative learning, but is not suitable for probabilistic learning. This hypothesis would be supported by evidence of a positive relationship between an enhanced MTL (N170) activation and declarative learning and a negative relationship between MTL activation and probabilistic learning in children with DLD. The FLP further suggests that striatal-based feedback processing (FRN) dominates and supports probabilistic learning in children with DLD. This hypothesis would be supported by a positive relationship between the FRN and probabilistic learning in children with this disorder.

## 2. Materials and Methods

### 2.1. Participants

Twenty-nine children from the Boston area participated in this study. Participants were recruited through printed and digital flyers distributed to local schools and clinics in the greater Boston area and through social media ads. Participants were classified as having either developmental language disorder (DLD; *n* = 14) or typical language development (TD; *n* = 15), based on the criteria described below. All participants were right-handed individuals between the ages of eight years one month and twelve years eight months (*M* = 10.62 years, *SD* = 1.42 years), with normal hearing, based on parental report, and normal or corrected vision; they reported no history of head injury or other neurological deficits, and English was their predominant language. All participants obtained a nonverbal intelligence score above the range of intellectual disability (SS > 80) on the Matrices subtest of the Kaufman Brief Intelligence Test, Second Edition [102].

Inclusion criteria for the DLD group were as follows: (a) Core Language Standard Score below 85 on the Clinical Evaluation of Language Fundamentals, Fifth Edition [103] or (b) Identification Core Score less than 34 if 8–11 years old, or less than 42 if 12–18 years old on the Test of Integrated Language and Literacy Skills [104]. One participant was missing the Core Language Score but met the criteria for the DLD group, with a standard score of 53 on the Receptive Language Index of the CELF-5. The TILLS was administered to six participants, five of whom did not meet criteria for DLD, based on CELF-5 performance, but whose parents reported their child had a history of language delay or impairment. All caregivers of children in the DLD group reported that their child had a history of delayed language development and persistent difficulties with spoken and/or written language. Inclusion criteria for the TD group were standard scores of 85 or above on all indices of the CELF-5 and no reported delays or difficulties with language. Both the CELF-5 and TILLS are measures commonly used by speech–language pathologists to diagnose children with DLD. The CELF-5 has a reported sensitivity and specificity of 0.97 for scores that are −1.33 below the mean [103]. The TILLS has a reported sensitivity range of 0.81–0.97 and a specificity of 0.81–1.00 for the age range of the participants in our study (8–12 years). The purpose of the TILLS is to measure skills at two language levels (sound/word and sentence/discourse levels) across oral- and written-language modalities (listening, speaking, reading, and writing). The kinds of tasks involved in this test require lexical knowledge, awareness of semantic relationships, representation of phonemic and morphemic components of novel words, reading, decoding, and written expression. The Identification Core Score on the TILLS is the sum of the standardized subtest scores that were found to be most discriminative for each age range [104].

Table 1 presents inclusionary data by group as well as results of one-way analyses of variance (ANOVA) and chi-squared analysis. The DLD and TD groups did not significantly differ in age or proportion of males and females. There were significant group differences favoring those with TD on both standardized assessments, as would be expected, given the classification criteria.

### 2.2. Procedure and Tasks

**Figure 1:**
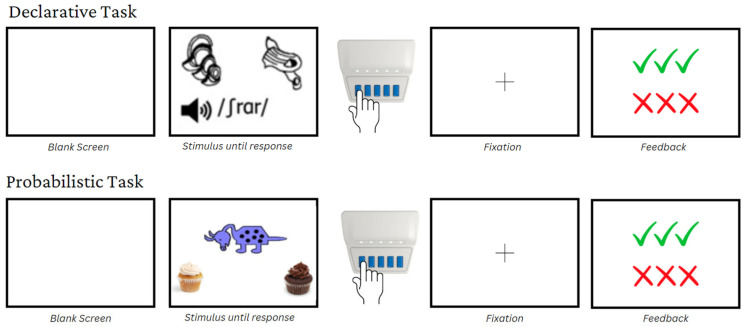
The two tasks resemble one another in terms of structure, and are differentiated by the type of learning required. The probabilistic task requires incremental learning of stimulus classification over many trials, whereas the declarative task requires trial-by-trial learning of each item.

Participants were enrolled in a larger study on feedback processing containing two-to-three sessions lasting 90 min each. Data collection occurred over two in-person sessions at the MGH Institute of Health Professions in Boston, followed by an online remote follow-up test on a computer. All participants completed a declarative learning task and a probabilistic learning task. Both tasks involved a feedback-based training phase immediately followed by a feedback-free test phase. The tasks differed in the type of stimuli to be learned. See Figure 1 for a visual illustration of each task. The study received approval from the Mass General Brigham Institutional Review Board (IRB). Parental signed consents and participant assents were obtained before data collection was initiated. Participants received monetary compensation for their participation.

#### 2.2.1. Declarative Task

In the declarative learning task, children were tasked with learning the correct associations between nonword names and novel objects (for a full description, see Arbel et al. 2021 [1]). During the training phase, participants were presented with 14 names of novel objects repeated in random order once in each of eight blocks, for a total of eight presentations of each name–object pairing and 112 total trials. Each trial began with a blank screen for 500 ms, followed by a display of two novel objects on a computer monitor and an auditory presentation of a name delivered through speakers. Children were tasked with selecting on a trial-by-trial basis the object associated with the spoken name, by pressing one of two buttons on a Chronos response box with either their left or right hand. The position of the objects on the screen (left or right) was randomized across trials. Responses were followed by a fixation cross for 1000 ms and then performance feedback for 1500 ms, indicating the accuracy of the pairing on a trial-by-trial basis. Among the pair of novel objects, one was a distractor with no associated name.

During the test phase, children were presented with the 14 pairs of objects and nonword names again. The trial structure was similar to the training, but no performance feedback was provided during the test. On a trial-by-trial basis, participants were tasked with selecting the correct novel object associated with a nonword name by pressing one of two buttons on a Chronos response box.

#### 2.2.2. Probabilistic Task

A simplified version of a probabilistic learning paradigm designed by Zeithamova et al. [105] was used. For a complete description of the task, please see Baron et al., 2023 [2] or Gul et al., 2022 [3]. Children were tasked with classifying novel cartoon animals into two categories, each defined by a combination of five binary features: head position, tail shape, foot shape, body shape, and body pattern. Each of the five features had two variations, linked in a probabilistic manner to one of the two classification categories. The classification outcomes were determined by the probabilistic combination of the five features. During the training phase, participants were presented with eight novel animal exemplars repeated in a random order five times in each of four blocks, for a total of 20 presentations of each exemplar and 160 total trials. Each trial started with a blank screen for 500 ms, followed by a visual display of an animal exemplar in the center of the screen, with the two category options at the bottom of the screen. Participants were instructed to classify each animal into one of two “families” by pressing one of two buttons on a Chronos response box with either their left or right hand. Each stimulus was displayed on the screen until a response was registered. Responses were followed by a fixation cross for 500 ms and then performance feedback for 1500 ms, indicating the correctness of the classification on a trial-by-trial basis. During the test phase, children were asked to classify a set of sixteen cartoon animals, eight of which were shown during the training phase and eight new animals that shared the same feature probabilities. Each animal was presented twice, for a total of 32 trials. Additionally, children were presented with the two prototypes that were not presented during the training phase. The prototypes were presented four times each throughout the test. In total, the test phase consisted of 40 trials presented in random order in a single block.

The trial structure was similar to the training, except participants did not receive any performance feedback during the test.

#### 2.2.3. Analysis of Behavioral Data

For each task, overall accuracy was examined during training and testing. Accuracy during feedback-based training was considered task-performance accuracy, and was an average of all training blocks. Accuracy on the feedback-free test measured learning outcomes. To evaluate response to feedback at the behavioral level, stay and switch measures were collected during the training phase of the two feedback-based learning tasks [1]. The stay measure was calculated as the proportion of repeated correct selections following positive feedback, and the switch measure was calculated as the proportion of trials that received negative feedback and were followed by a change in selection/response. The stay measure served to signify an effective response to positive feedback, while the switch measure served to reflect an effective response to negative feedback.

Behavioral data were analyzed using a series of mixed analyses of variance (ANOVA). For analyses of accuracy, the dependent variable was the percentage of correct trials. For analyses of response to feedback during training, the dependent variable was the proportion of each trial type (i.e., switch or stay). The between-subjects variable was the group (TD and DLD), and the within-subjects variable was either the phase (training and test) or response type (switch and stay). When indicated by a significant interaction effect, pairwise comparisons were analyzed using the Bonferroni correction.

#### 2.2.4. EEG Data Acquisition and Processing

EEG data were continuously recorded using the GES 400 System by Electrical Geodesics Inc. (EGI, Eugene, OR, USA) with 32-channel HydroCel Geodesic Sensor Nets from EGI. The design of the experiment and the presentation of stimuli were facilitated through the use of programmable experiment generation software (E-prime 2.0; Psychological Software Tools, Pittsburgh, PA, USA). EEG data were recorded at a sampling rate of 1000 Hz, using the vertex electrode as the reference [106] and keeping impedances below 50 kΩ. Offline signal processing was carried out using custom MATLAB (The MathWorks, Inc., Natick, MA, USA) scripts in conjunction with the open-source EEGLAB toolbox [107]. EEG data were resampled at 250 Hz, filtered using a high-pass finite impulse response (FIR) at 0.1 Hz and a low-pass 40 Hz filter, and then re-referenced to the average activity of all electrodes [108]. The EEGLAB toolbox was used to extract data for the FRN from the fronto-central FCz electrode and for the N170 from the left parietal P7 for each task, participant, and condition. The FCz and P7 electrodes were chosen based on a visual inspection of the topo maps and on previous findings that the FRN signal is strongest at FCz [1,3,87,109,110,111] and the N170 signal is strongest at P7 [82,96]. Each trial was visually inspected for movement artifacts, which were manually removed. An Adaptive Mixture ICA (AMICA) was applied separately to every single subject dataset [112], to detect and correct eye movement and eye blinks. The data were then sorted into two feedback categories: negative and positive feedback. EEG epoched data were analyzed separately for time and time–frequency domains.

#### 2.2.5. ERP Analysis

The processed signals underwent segmentation into epochs of 1200 ms duration, extending to 200 ms prior to and 1000 ms following the presentation of feedback. Subsequently, baseline correction was applied to the averaged data, utilizing the signal within the 200 ms timeframe preceding the feedback stimulus, denoted as the interval from −200 to 0 ms. To extract and measure the ERP components of interest, the ERP datasets were subjected to Temporal Principal Component Analysis (TPCA) as a means of reducing temporal dimensionality and distinguishing overlapping ERP components. The TPCA procedure was facilitated through Promax rotation, as previously demonstrated in the existing literature [3,87,96,113,114]. To measure the FRN ERP component, TPCA was conducted on electrode FCz, yielding seven temporal factors for the data extracted from the probabilistic paradigm and nine temporal factors for the declarative task. In the probabilistic task, Temporal Factor 5 (TF5), which peaked at around 250 ms, was identified as capturing the FRN activation. The activations corresponding to the FRN in the declarative task were also identified as being captured by TF5. To measure the N170 ERP components, TPCA was conducted on electrode P7, yielding a set of eight temporal factors for the probabilistic task and seven factors for the declarative task. Temporal Factor 2 (TF2) in the probabilistic task and TF6 in the declarative task were identified as capturing the N170 component.

#### 2.2.6. Time–Frequency Analysis

The EEG data analysis encompassed several steps to examine the temporal and spectral characteristics of brain activity during two specific tasks. Initially, time–frequency decomposition of EEG signals was performed utilizing customized MATLAB scripts in conjunction with the EEGLAB toolbox [107]. Continuous EEG recordings from the FCz and P7 electrodes obtained from each participant, feedback type, and task were segmented into 3 s epochs, spanning from 1000 ms preceding to 2000 ms following the onset of feedback. To effectively balance temporal- and frequency-resolution considerations, the ‘newtimef()’ function was employed, which applies the Morlet wavelet transform to the epoch time-series data, dynamically adapting the wavelet with increasing frequencies [107]. Consequently, utilizing ‘newtimf()’, the average power was computed across 90 linearly spaced frequencies ranging from 1 to 30 Hz, segmented into 300 linearly spaced time bins (ranging from 1 cycle at the lowest frequency to 15 cycles at the highest). Each epoch was subsequently temporally adjusted, reducing its duration to −447 ms before and +1447 ms after the feedback onset. We extracted measures of Event-Related Spectral Perturbation (ERSP) and Inter-Trial Coherence (ITC) within the delta frequency range (1 to 3 Hz) and theta frequency range (4 to 7 Hz) at FCz, using time windows that correspond to the FRN (200–400 ms) component [85,86,87]. A time window of 150 to 250 ms at P7 was used for the time–frequency analysis of the N170 [82,96]. ERSP and ITC values were averaged across trials for each participant.

#### 2.2.7. Statistical Analysis of EEG Data

Factor scores of the ERP components of interest (FRN, N170), as well as power and ITC values of delta and theta in the FRN and N170 windows obtained from each participant and task, were subjected to mixed ANOVAs with group (TD, DLD) as a between-subject variable, and feedback valence (positive feedback, negative feedback) as a within-subject variable. Separate analyses were performed for each task. Significant effects were corrected for non-sphericity using Greenhouse–Geisser corrections, and are reported with the corrected degrees of freedom when appropriate. To evaluate the potential relationship between feedback-related activation at the electrophysiological level and learning, Pearson correlational analyses were performed between the behavioral measures of learning (task performance and learning outcomes) and electrophysiological data (ERP and ITC data). *p*-values were adjusted for multiple correlations using the Benjamini–Hochberg procedure to control the false discovery rate (FDR). Statistical analysis employed IBM SPSS Statistics 24.0 (IBM, Armonk, NY, USA).

## 3. Results

### 3.1. Behavioral Data

Table 2 presents behavioral data for each task and group.

#### 3.1.1. Declarative Learning Task

To evaluate declarative task performance and learning outcomes, a mixed ANOVA with the group (TD and DLD) as a between-subjects variable and the phase (training and test) as a within-subjects variable was conducted. There was a significant main effect of group, *F*(1, 27) = 6.23, *p* = 0.019, *η_p_*^2^ = 0.187, indicating that accuracy was higher for children in the TD group than for children in the DLD group, in both phases. There was also a significant main effect of phase, *F*(1, 27) = 70.59, *p* < 0.001, *η_p_*^2^ = 0.723, in which test accuracy was significantly higher than training accuracy for both groups. The interaction between group and phase was not significant.

To evaluate stay and switch responses during the training phase of the declarative task, a mixed ANOVA with group (TD and DLD) as a between-subjects variable and response type (stay and switch) as a within-subjects variable was conducted. There was a significant main effect of response type, *F*(1, 27) = 36.30, *p* < 0.001, *η_p_*^2^ = 0.573, but not group, *F*(1, 27) = 2.75, *p* = 0.109, *η_p_*^2^ = 0.092. These effects were qualified by a significant interaction between group and response type, *F*(1, 27) = 7.38, *p* = 0.011, *η_p_*^2^ = 0.215. Both groups demonstrated a significantly larger proportion of stay responses than switch responses (TD *p* < 0.001; DLD *p* = 0.029). Groups did not differ in their proportion of switch responses (*p* = 0.757), but the TD group demonstrated a significantly larger proportion of stay responses than the DLD group (*p* = 0.010).

#### 3.1.2. Probabilistic Learning Task

To evaluate probabilistic task performance and learning outcomes, a mixed ANOVA with the group (TD and DLD) as a between-subjects variable and the phase (training and test) as a within-subjects variable was conducted. There was a significant main effect of group, *F*(1, 27) = 8.75, *p* = 0.006, *η_p_*^2^ = 0.245, indicating that accuracy was higher for children in the TD group than for children in the DLD group, in both phases. There was also a significant main effect of phase, *F*(1, 27) = 11.05, *p* = 0.003, *η_p_*^2^ = 0.290, in which test accuracy was significantly higher than training accuracy for both groups. The interaction between group and phase was not significant.

To evaluate stay and switch responses during the training phase of the probabilistic task, a mixed ANOVA with group (TD and DLD) as a between-subjects variable and response type (stay and switch) as a within-subjects variable was conducted. There was a significant main effect of response type, *F*(1, 27) = 15.94, *p* < 0.001, *η_p_*^2^ = 0.371, but not group, *F*(1, 27) = 2.25, *p* = 0.145, *η_p_*^2^ = 0.077. These effects were qualified by a significant interaction between group and response type, *F*(1, 27) = 9.05, *p* = 0.006, *η_p_*^2^ = 0.251. The TD group demonstrated a significantly larger proportion of stay responses than switch responses (*p* < 0.001), while the DLD group demonstrated an equal amount of each response type (*p* = 0.500). Groups did not differ in their proportion of switch responses (*p* = 0.457), but the TD group demonstrated a significantly larger proportion of stay responses than the DLD group (*p* = 0.016).

#### 3.1.3. Summary of Behavioral Results

The results of the behavioral analyses indicate that on both the declarative and probabilistic learning tasks, children with DLD demonstrated poorer task performance, lower learning outcomes, and reduced proportion of stay responses when compared with their age-matched peers with TD. In both learning paradigms, children with DLD and TD were better at repeating correct responses following positive feedback than they were at switching responses following negative feedback. It is important to note that the goal of the project was not to conduct a task-performance comparison, because the tasks cannot be reliably controlled for difficulty level or reliance on cognitive resources.

### 3.2. Feedback-Related Electrophysiological Data

**Figure 2:**
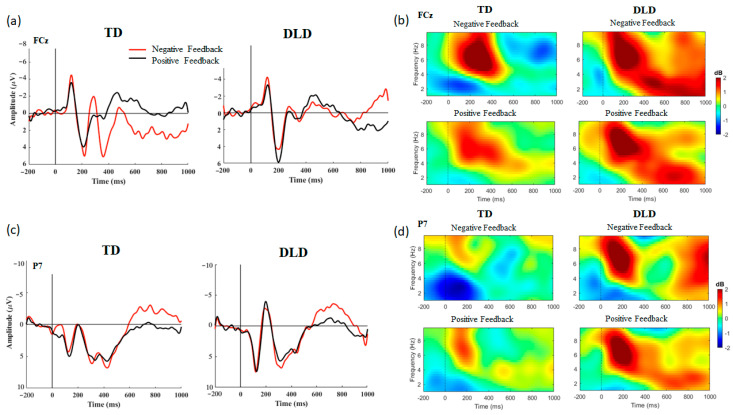
Event-related potentials (left (**a**,**c**)) and time–frequency power measures (right (**b**,**d**)) measured from electrodes FCz (top (**a**,**b**)) to capture the feedback-related negativity (FRN), and P7 (bottom (**c**,**b**)) to capture the N170 during a declarative learning task performed by children with typical development (TD) and children with developmental language disorder (DLD). Activation was measured in response to negative- and positive-feedback presentations.

**Figure 3:**
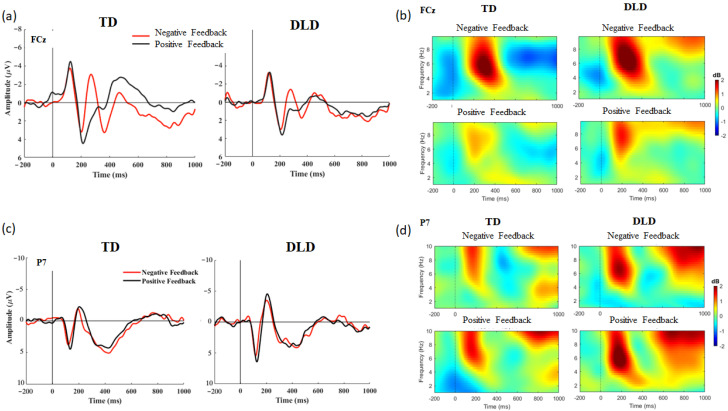
Event-related potentials (left (**a**,**c**)) and time–frequency power measures (right (**b**,**d**)) measured from electrodes FCz (top (**a**,**b**)) to capture the feedback-related negativity (FRN), and P7 (bottom (**c**,**b**)) to capture the N170 during a probabilistic learning task performed by children with typical development (TD) and children with developmental language disorder (DLD). Activation was measured in response to negative- and positive-feedback presentations.

To examine differences in neural activation associated with feedback processing between the two groups (i.e., TD and DLD), a mixed ANOVA with feedback valence as a within-subject variable and group as a between-subject variable was conducted on the amplitudes of the FRN and N170 for each learning task. Figure 2 presents the FRN and N170 components elicited during the declarative learning task and analyzed for the time and time–frequency levels for each group (TD, DLD). Figure 3 shows the FRN and N170 components elicited during the probabilistic task and analyzed for the time and time–frequency levels for each group. Results of the correlational analyses between the behavioral and electrophysiological data are presented in Table 3 and Table 4.

#### 3.2.1. Declarative Learning Task

FRN. A mixed ANOVA revealed no significant group effect on the amplitude of the FRN component, *F*(1, 27) = 0.617, *p* = 0.439, *η_p_*^2^ = 0.022. Additionally, there was no significant main effect of feedback valence, *F*(1, 27) = 0.023, *p* = 0.882, *η_p_*^2^ = 0.001. However, an interaction between feedback and group was identified, *F*(1, 27) = 4.474, *p* = 0.044, *η_p_*^2^ = 0.142. Post hoc *t*-tests revealed that the FRN differences between negative and positive feedback were larger in the TD group when compared to the DLD group, *t* (27) = 2.115, *p* = 0.044. Evaluation of theta power in the FRN time window revealed no group effect, *F*(1, 27) = 0.113, *p* = 0.739, *η_p_*^2^ = 0.004, feedback valence effect, *F*(1, 27) = 3.275, *p* = 0.081, *η_p_*^2^ = 0.108, or feedback-by-group interaction, *F*(1, 27) = 1.731, *p* = 0.199, *η_p_*^2^ = 0.06, indicating that while theta activation was strong during feedback processing, it did not differ between groups and feedback valence. Examination of theta ITC within the FRN time window revealed a significant effect of feedback valence, *F*(1, 27) = 14.442, *p* < 0.001, *η_p_*^2^ = 0.348, indicating more consistent theta activation across trials of negative feedback than positive feedback. There was no significant group effect, *F*(1, 27) = 0.056, *p* = 0.814, *η_p_*^2^ = 0.002 or feedback-by-group interaction, *F*(1, 27) = 3.260, *p* = 0.082, *η_p_*^2^ = 0.108. Evaluation of delta power within the FRN time window revealed no group effect, *F*(1, 27) = 3.201, *p* = 0.085, *η_p_*^2^ = 0.106, feedback valence effect, *F*(1, 27) = 0.224, *p* = 0.639, *η_p_*^2^ = 0.008, or interaction between group and feedback valence, *F*(1, 27) = 0.464, *p* = 0.430, *η_p_*^2^ = 0.022. Delta ITC within the same time window exhibited no significant effects related to group, *F*(1, 27) = 0.412, *p* = 0.526, *η_p_*^2^ = 0.015, feedback valence, *F*(1, 27) = 0.865, *p* = 0.360, *η_p_*^2^ = 0.031, or group-by-feedback interaction, *F*(1, 27) = 0.678, *p* = 0.417, *η_p_*^2^ = 0.024.

FRN and Performance. A larger (more negative) amplitude of the FRN component in response to negative feedback was found to be associated with better task performance in the TD group, *r*(15) = −0.661, *p* = 0.022. Additionally, within the TD group, higher theta ITC in response to negative feedback was associated with better task performance, *r*(15) = 0.691, *p* = 0.013. The association between theta ITC in response to negative feedback and learning outcomes approached significance, *r*(15) = 0.565, *p* = 0.056. FRN elicited by children with DLD did not exhibit correlations with performance (*p* > 0.05). However, higher delta ITC in response to positive feedback in the DLD group was found to be associated with task performance, *r* (14) = 0.601, *p* = 0.028, and learning outcomes, *r*(14) = 0.601, *p* = 0.028.

N170. A significant main effect of group was identified for the N170 component, *F*(1, 27) = 6.846, *p* = 0.014, *η_p_*^2^ = 0.202, revealing that children with DLD elicited larger negative amplitudes overall, as compared to their TD peers. There was no significant main effect of feedback valence, *F*(1, 27) = 0.050, *p* = 0.825, *η_p_*^2^ = 0.002 or interaction between group and feedback, *F*(1, 27) = 0.043, *p* = 0.837, *η_p_*^2^ = 0.002. Evaluation of theta power within the N170 time window revealed a significant group effect, *F*(1, 27) = 10.473, *p* = 0.003, *η_p_*^2^ = 0.279, with larger theta power in the DLD than in the TD group. There was no feedback valence effect, *F*(1, 27) = 2.021, *p* = 0.167, *η_p_*^2^ = 0.070 or feedback-by-group interaction, *F*(1, 27) = 2.091, *p* = 0.160, *η_p_*^2^ = 0.072. Examination of theta ITC revealed no significant group effect, *F*(1, 27) = 3.558, *p* = 0.070, *η_p_*^2^ = 0.116, indicating that although theta power was stronger in the DLD group, there was only a trend for theta power to be consistently higher in the DLD group across trials when it was compared to the TD group. A feedback valence effect was found, *F*(1, 27) = 5.267, *p* = 0.030, *η_p_*^2^ = 0.163, indicating that theta power was more consistent across trials in response to negative feedback than to positive feedback. No interaction between group and feedback valence was found, *F*(1, 27) = 0.113, *p* = 0.740, *η_p_*^2^ = 0.004. Evaluation of delta power within the N170 time window revealed no significant group effect, *F*(1, 27) = 2.252, *p* = 0.145, *η_p_*^2^ = 0.077, feedback valence effect, *F*(1, 27) = 3.226, *p* = 0.084, *η_p_*^2^ = 0.002 or interaction between group and feedback valence, *F*(1, 27) = 0.920, *p* = 0.346, *η_p_*^2^ = 0.00. While there was no group effect for delta ITC, *F*(1, 27) = 0.734, *p* = 0.399, *η_p_*^2^ = 0.026, a significant feedback valence effect was revealed *F*(1, 27) = 4.957, *p* = 0.035, *η_p_*^2^ = 0.155, indicating that delta power was more consistent across trials of negative feedback than trials of positive feedback. There was no interaction between group and feedback valence for delta ITC, *F*(1, 27) = 0.128, *p* = 0.723, *η_p_*^2^ = 0.005.

N170 and Performance. No significant correlations between performance and delta and theta power in the N170 time window were found (*ps* > 0.05). When evaluating the relationship between the ITC measure and performance in the DLD group, higher delta ITC in response to negative feedback in the N170 window was associated with better task performance, *r*(14) = 0.641, *p* = 0.040, and learning outcomes, *r*(14) = 0.705, *p* = 0.031. Such correlations were not observed in children with TD.

Summary of electrophysiological findings in the declarative task: FRN differences between negative and positive feedback were found to be larger in the TD group when compared to the DLD group. Both groups exhibited more consistent theta activation across trials of negative feedback than in trials of positive feedback. However, only the TD group showed an association between a large FRN and stronger theta in response to negative feedback and task performance. In the DLD group, stronger delta ITC in response to positive and negative feedback in the FRN window was associated with better learning outcomes. The N170 was found to be larger in the DLD group. Stronger delta ITC in response to negative feedback in the N170 window was associated with better learning in the DLD group only.

#### 3.2.2. Probabilistic Learning Task

FRN. A significant group effect was detected, *F*(1, 27) = 4.681, *p* = 0.040, *η_p_*^2^ = 0.148, indicating that the FRN amplitude was larger in the TD group than in the DLD group. A main effect of feedback valence was also found, *F*(1, 27) = 35.521, *p* < 0.001, *η_p_*^2^ = 0.568, confirming that negative feedback was associated with a larger (more negative) amplitude than positive feedback. Moreover, a significant interaction between group and feedback valence was identified, *F*(1, 27) = 8.345, *p* = 0.008, *η_p_*^2^ = 0.236. A follow-up *t*-test revealed that FRN in response to negative feedback was larger in children with TD compared to children with DLD, *t*(14) = 2.865, *p* = 0.008, while there were no differences between the two groups in the FRN amplitude in response to positive feedback, *t*(14) = 0.672, *p* = 0.507. Evaluation of theta power within the FRN time window revealed no group effect, *F*(1, 27) = 0.005, *p* = 0.944, *η_p_*^2^ = 0. A main effect of feedback valance was found, *F*(1, 27) = 14.479, *p* < 0.001, *η_p_*^2^ = 0.349, indicating stronger theta in response to negative than to positive feedback. There was no feedback by group interaction, *F*(1, 27) = 0.005, *p* = 0.943, *η_p_*^2^ = 0. When theta ITC was examined, no group effect, *F*(1, 27) = 0.011, *p* = 0.918, *η_p_*^2^ = 0, or feedback main effect, *F*(1, 27) = 4.06, *p* = 0.054, *η_p_*^2^ = 0.131 were detected. However, there was a trend for higher theta ITC in response to negative than positive feedback. There was no feedback by group interaction, *F*(1, 27) = 0.562, *p* = 0.460, *η_p_*^2^ = 0.20. Evaluation of delta power revealed no group effect, *F*(1, 27) = 0, *p* = 0.999, *η_p_*^2^ = 0, feedback valence effect, *F*(1, 27) = 0.02, *p* = 0.889, *η_p_*^2^ = 0.001, or interaction between group and feedback valence, *F*(1, 27) = 0.001, *p* = 0.979, *η_p_*^2^ = 0. Delta ITC exhibited no significant effects related to group, feedback valence, or group-by-feedback interaction (*p* > 0.05).

FRN and Performance. A negative correlation between the amplitudes of the FRN component in response to positive feedback and learning outcomes in the TD group was non-significant, *r*(15) = −0.531, *p* = 0.126. A positive correlation in the TD group between higher delta ITC in the FRN time window in response to negative feedback and task performance approached significance, *r*(15) = 0.652, *p* = 0.051. In the DLD group, no significant correlations were found.

N170. While there was a trend for the N170 amplitude to be larger in the DLD group, these differences did not reach significance, *F*(1, 27) = 3.675, *p* = 0.066, *η_p_*^2^ = 0.120. A significant main effect of feedback valence was identified, *F*(1, 27) = 6.839, *p* = 0.014, *η_p_*^2^= 0.202, indicating that positive feedback was associated with a larger N170 than negative feedback. There was no interaction effect between group and feedback valence, *F*(1, 27) = 2.160, *p* = 0.153, *η_p_*^2^ = 0.074. Evaluation of theta power revealed no group effect, *F*(1, 27) = 2.147, *p* = 0.154, *η_p_*^2^ = 0.074, feedback valence effect, *F*(1, 27) = 2.984, *p* = 0.095, *η_p_*^2^ = 0.100, or interaction between group and feedback valence, *F*(1, 27) = 0.125, *p* = 0.727, *η_p_*^2^ = 0.005. Similarly, examination of theta ITC resulted in no group effect, *F*(1, 27) = 1.390, *p* = 0.249, *η_p_*^2^ = 0.049, feedback valence effect, *F*(1, 27) = 0.839, *p* = 0.368, *η_p_*^2^ = 0.030, or interaction between group and feedback valence, *F*(1, 27) = 0.090, *p* = 0.766, *η_p_*^2^ = 0.003 being detected. An evaluation of delta power within the N170 time window revealed no statistically significant group effect, *F*(1, 27) = 0.362, *p* = 0.552, *η_p_*^2^ = 0.013, or feedback valence effect, *F*(1, 27) = 0.160, *p* = 0.692, *η_p_*^2^ = 0.006. The interaction between group and feedback valence was not significant, *F*(1, 27) = 4.179, *p* = 0.051, *η_p_*^2^ = 0.134, but showed a trend that may be linked to a stronger delta power in response to positive than in response to negative feedback in the DLD group, in comparison to the TD group. An evaluation of the delta ITC resulted in no group effect, *F*(1, 27) = 0.231, *p* = 0.635, *η_p_*^2^ = 0.008, feedback valence effect, *F*(1, 27) = 0.129, *p* = 0.722, *η_p_*^2^ = 0.005, or interaction between group and feedback valence, *F*(1, 27) = 0.121, *p* = 0.731, *η_p_*^2^ = 0.004.

N170 and Performance. No significant correlations were observed between the N170 ERP component and performance in the two groups. A negative correlation was found for children in the TD group between N170 theta ITC in response to both negative and positive feedback and task performance, *r*(15) = −0.625, *p* = 0.048, *r*(15) = −0.780, *p* = 0.009, respectively. There were no correlations between performance and delta and theta activity in the DLD group (*p* > 0.05).

Summary of electrophysiological findings in the probabilistic task: FRN in response to negative feedback was larger in children with TD compared to children with DLD. Stronger and more consistent theta in response to negative feedback in the FRN window was found across groups. In the TD group only, stronger delta power in response to negative feedback in the FRN window was associated with better task performance. No group differences were found in the N170 ERP component or in its low frequencies. In the TD group, stronger theta in the N170 window was associated with poorer task performance and learning outcomes.

#### 3.2.3. Summary of Results

In both tasks, children with TD achieved better learning outcomes than children with DLD. Behavioral measures of feedback processing indicated that both groups were inefficient in processing negative feedback and that children with TD were better than children with DLD at repeating correct responses after positive feedback, in both tasks. Electrophysiological measures indicated that FRN amplitude in response to negative feedback was larger in children with TD, in both tasks. In the declarative task, larger FRN and stronger theta in response to negative feedback in the TD group were associated with better learning. In the DLD group, on the other hand, it was the contribution of delta to the FRN that was associated with task performance and learning outcomes. In the probabilistic task, higher delta ITC in response to negative feedback in the FRN window was associated with better learning only in the TD group. The N170 ERP component, which may reflect the activation of MTL-based declarative learning mechanisms, was found to be larger in the DLD group, but only in the declarative learning task. Children in the DLD group showed stronger and consistent N170 theta in response to negative feedback in the declarative task. A stronger delta N170 in response to negative feedback was found to be associated with better learning in this group. In the TD group, no correlations were found between the N170 and performance in the declarative task. In the probabilistic task, on the other hand, lower theta N170 in response to both positive and negative feedback in the TD group was associated with better learning.

In summary, differences in feedback-based learning and feedback processing were found between the groups in the two learning paradigms. Children with DLD achieved poorer learning outcomes in both tasks, and did not show the same level of striatal-based response to feedback as their peers. Interestingly, the data of children in the DLD group were consistent with the proposed FLP theory in the context of declarative learning, such that better learning outcomes were associated with larger N170. In the TD group, however, support for the theory comes from the probabilistic learning task, in which stronger FRN delta activation, which is assumed to be generated in the striatum, was associated with better learning, and stronger MTL-based N170 was linked to poorer learning outcomes.

## 4. Discussion

The primary aim of this study was to examine whether the process of feedback-based learning in children with DLD varies, based on the unique feedback-processing demands dictated by the learning paradigm. To achieve this, we examined response to feedback on the behavioral and electrophysiological levels in children with and without DLD within the context of declarative and probabilistic learning paradigms in which feedback processing demands differ. We examined the striatum-based FRN and MTL-related N170 ERP components and their frequency composition in relation to learning performance and outcomes. We evaluated our results in light of competing hypotheses, including a novel parity theory, according to which feedback processing in children with DLD is improved when it is governed by the same system dominating the learning process. More specifically, we hypothesized that in children with DLD, greater MTL-based (N170) feedback processing will be associated with better declarative learning but poor probabilistic learning, and that striatum-based FRN will support probabilistic learning. The results were also evaluated in light of the PDH, which states that declarative resources serve to compensate for impaired striatal-based learning in DLD.

Our results highlight various behavioral and neural response patterns differentiating children with DLD from age-matched peers with TD. Children with DLD demonstrated poorer learning outcomes in both learning paradigms, compared with their TD peers. When evaluating the switch (changing a response following negative feedback) and stay (i.e., keeping a response following positive feedback) behavioral responses to feedback, our results indicated that switch behaviors were equally inconsistent across groups and paradigms. These results indicate that children with and without DLD are inefficient in using negative feedback to advance their learning. These findings are in line with previous reports of children showing better learning outcomes under errorless or feedback-free conditions (see [1,115]), and can be explained by the cognitive demands that feedback processing places on a learner, regardless of the learning conditions. More specifically, whether the feedback is deterministic or probabilistic, it leads to the application of conscious, intentional, hypothesis-driven processes that rely on explicit mechanisms, including working memory and other executive functions that are still developing during the school years (see [116,117,118,119,120,121]), and known to be limited in children with DLD [5,6,7,8,9,10,11,12,13]. Our results indicated equally inefficient processing of negative feedback in both groups, and an advantage in the processing of positive feedback among children with TD. Poor usage of negative feedback by children has been previously reported [122,123,124], and is likely stemming from the processing load needed for the rejection of a current hypothesis, the acceptance of an alternative hypothesis, and the updating of memory with the correct information. Our findings indicate that stay behaviors were more prevalent among children with TD in the two learning paradigms when compared with the DLD group. These findings may point to a notable difficulty among children with DLD in maintaining the reinforced associations or behaviors in memory over time.

Electrophysiological data revealed additional group and task-specific similarities and differences in feedback processing and feedback-based learning. Similar to children with TD, the DLD group displayed evidence of a greater response to negative feedback when compared to positive feedback. However, in both paradigms, differences in processing of positive and negative feedback in the DLD group were less evident than those observed in the TD group, indicating limitations in distinguishing between the two types of feedback to guide performance. These observations are further enlightened by the results of the frequency decomposition analysis of the FRN. More specifically, in both tasks, children with and without DLD exhibited increased theta activity within the FRN time range in response to negative feedback, which aligns with previous research emphasizing the significance of theta oscillations in feedback processing [85,87,91]. However, only the TD group displayed a clear relationship between stronger FRN in response to negative feedback and better learning outcomes in both learning paradigms.

To evaluate the involvement of the MTL in feedback-based learning in the two groups and paradigms, N170 was examined in relation to learning. Our results indicate that the N170 was larger in the DLD group, but only in the declarative learning task. These observations support the contention that children with DLD rely more heavily on the MTL system for declarative feedback-based learning. The behavioral and electrophysiological data of the present study can shed light on the ongoing debate about the underlying impaired learning mechanism in DLD. To support the PDH, our data should have indicated impaired performance by children with DLD in the probabilistic task and comparable performance to their peers in declarative learning. The PDH further suggests that declarative learning serves as a compensatory learning mechanism. To support this argument, the N170 should be enhanced in the DLD group, particularly in the probabilistic learning task, and be associated with improved learning outcomes. The results of the present study do not support the PDH. First, while children with DLD performed poorly on the probabilistic task, they also demonstrated difficulties achieving comparable learning outcomes in the declarative task. Second, the N170 was indeed larger in the DLD group, and was associated with better learning outcomes, but only in the context of declarative learning. The N170 was not found to be larger or associated with better learning outcomes when learning was probabilistic in nature. In other words, in children with DLD, MTL-based activation was associated with declarative learning and was beneficial for this type of learning. However, the MTL-based activation was not found to be associated with probabilistic learning or beneficial for this type of learning. Interestingly, enhanced N170 within the probabilistic learning context was associated with poorer learning outcomes in the TD group. These results suggest that MTL activation can be disruptive when learning should be dominated by the striatum. The study also evaluated whether in children with DLD reliance on declarative learning mechanisms disrupts learning. Our data do not provide evidence to support or refute the contention that children with DLD over rely on declarative learning mechanisms. More specifically, the N170 was not found to be enhanced during probabilistic learning in the DLD group, nor was it associated with poor performance of children with this disorder. Our proposed FLP theory suggests that learning in children with DLD is optimal when the system that dominates learning also dominates feedback processing. The present study provides some evidence in support of this theory. The enhanced N170 during the declarative learning task and its positive association with learning in the DLD group suggests that during declarative learning, the declarative learning system dominates feedback processing to the benefit of children with this disorder. However, the present report does not provide evidence that striatal-based feedback processing facilitates probabilistic learning in children with DLD.

Limitations—The study sample is relatively small, which impacts our ability to conduct more robust statistical analyses or make strong conclusions about the generalization of results. However, the participant groups were well-matched for chronological age and gender, ensuring that basic demographic characteristics did not influence results. While the groups differed in nonverbal intelligence, this variability in cognitive skills is an accurate representation of the broader population of children with DLD. Another limitation of this work is that the two task paradigms cannot be directly compared to one another. While the general format, task design, and trial structure are comparable, each paradigm contains a different type of stimuli and engages different cognitive resources. Therefore, it is essential to interpret the findings within each paradigm separately. By examining the evidence within each paradigm for patterns of behavioral response and electrophysiological processing, we can gain insights into learning and feedback-processing mechanisms in different environments.

## 5. Conclusions

The results of the reported study suggest that outcomes of declarative and probabilistic feedback-based learning were poorer among children with DLD when compared with their age-matched peers. Electrophysiological data highlight processing patterns that shed light on the unique learning profile of children with this disorder. More specifically, while the striatum-generated FRN was activated in both declarative- and probabilistic-feedback-based paradigms in both groups, it was smaller in the DLD group. Declarative learning in children with DLD was dominated and enhanced by the MTL-generated N170. In children with TD, increased MTL activation, as measured by the N170, was detrimental to probabilistic learning. The results provide some support for our feedback learning parity hypothesis, whereby feedback-based learning is enhanced when the system that dominates learning also dominates feedback processing. Future studies should further evaluate the possible connectivity limitation between the two learning systems (MTL and striatum) in children with DLD.

## Figures and Tables

**Table 1:** Group Comparison on Inclusionary Measures.

	TD	DLD	One-Way ANOVA Results		
Inclusionary Measures	*n* = 15	*n* = 14	*df*	*F*	*p*
Age (in months)	129.27 (15.00)	125.50 (19.27)	1, 27	0.35	0.560
KBIT-2 Matrices Score	113.13 (9.29)	103.79 (12.45)	1, 27	5.30	0.029 ^‡^
CELF-5 Core Language Score	106.73 (10.49)	84.08 (9.24)	1, 26	36.22	<0.001
TILLS Identification Core Score *	--	70.33 (10.93)	--	--	--
			Chi-Squared Test Results		
Sex:			*df*	*X* ^2^	*p*
Female	5	6	1, *n* = 29	0.056	0.812
Male	9	9			

Note. Values are presented as mean (standard deviation); assessment data are standard scores. KBIT-2 = Kaufman Brief Intelligence Test, 2nd Edition; CELF-5 = Clinical Evaluation of Language Fundamentals, 5th Edition; TILLS = Test of Integrated Language and Literacy Skills. * Six participants in the DLD group completed the supplemental TILLS assessment. ^‡^ Note that, although within the normal limits, nonverbal IQ scores of children with DLD in this sample are lower than those of children with TD. Non-verbal IQ scores are typically lower in children with DLD than their peers because the disorder is characterized by limitations in cognitive resources such as attention and working memory.

**Table 2:** Behavioral Data by Task and Group.

	Declarative		Probabilistic	
	TD (*n* = 15)	DLD (*n* = 14)	TD (*n* = 15)	DLD (*n* = 14)
Training Accuracy ^a^	72 (12)	61 (13)	60 (8)	55 (8)
Test Accuracy ^a^	89 (15)	74 (16)	68 (11)	59 (5)
Stay Responses ^b^	70 (8)	58 (14)	62 (9)	53 (10)
Switch Responses ^c^	48 (10)	50 (10)	49 (7)	51 (6)

Note. Values are presented as mean (standard deviation). ^a^ Accuracy was measured as percentage correct. ^b^ Stay response is the proportion of positive feedback trials followed by a stay response (i.e., repeating the response for a specific stimulus). ^c^ Switch behavior was defined as the proportion of negative feedback trials followed by a switch response (i.e., choosing a different response for a specific stimulus).

**Table 3:** Correlations between the FRN and N170 Event-Related Potentials (ERPs) and learning measures (performance during the training phase and learning outcomes on the test) for the two groups (TD, DLD) and tasks (Declarative, Probabilistic).

	Declarative Task ERP				Probabilistic Task ERP			
	TD		DLD		TD		DLD	
	Learning Outcomes	Task Performance	Learning Outcomes	Task Performance	Learning Outcomes	Task Performance	Learning Outcomes	Task Performance
FRN to NF	−0.494	−0.661 *	−0.386	−0.245	−0.401	0.126	−0.164	0.31
	0.092	0.022	0.347	0.597	0.207	0.786	0.69	0.56
FRN to PF	−0.101	−0.155	−0.042	0.184	−0.531	0.005	0.181	0.41
	0.721	0.696	0.886	0.636	0.126	0.986	0.69	0.436
N170 to NF	−0.186	0.038	−0.342	−0.375	0.296	0.037	−0.122	0.083
	0.783	0.892	0.348	0.348	0.341	0.897	0.824	0.824
N170 to PF	−0.199	−0.111	−0.038	−0.139	0.486	0.366	−0.065	0.324
	0.783	0.834	0.899	0.762	0.133	0.27	0.824	0.777

Note. *p*-values are adjusted for multiple comparisons using the False Discovery Rate (FDR) correction method. NF = negative feedback; PF = positive feedback; * *p* < 0.05.

**Table 4:** Correlations between the delta and theta Inter-Trial Coherence (ITC) within the FRN and N170 ranges and learning measures (performance during the training phase and learning outcomes on the test) for the two groups (TD, DLD) and tasks (declarative, probabilistic). * *p* < 0.05, ** *p* < 0.01.

		TD Declarative		DLD Declarative		TD Probabilistic		DLD Probabilistic	
		Learning Outcomes	Task Performance	Learning Outcomes	Task Performance	Learning Outcomes	Task Performance	Learning Outcomes	Task Performance
FRN	Delta to NF	0.344	0.343	0.611 *	0.626 *	0.312	0.652	−0.390	0.003
		0.419	0.420	0.028	0.027	0.515	0.051	0.839	0.990
	Delta to PF	−0.136	−0.009	0.601 *	0.515	−0.241	0.114	0.170	−0.207
		0.755	0.975	0.028	0.059	0.581	0.824	0.839	0.839
	Theta to NF	0.565	0.691 *	0.365	0.206	0.522	0.294	−0.232	0.438
		0.056	0.013	0.452	0.719	0.189	0.346	0.638	0.235
	Theta to PF	0.049	−0.037	−0.006	−0.120	−0.037	−0.293	−0.504	0.057
		0.898	0.898	0.984	0.819	0.897	0.346	0.198	0.847
N170	Delta to NF	0.195	0.261	0.705 *	0.641 *	0.008	0.059	0.006	0.350
		0.664	0.584	0.024	0.040	0.976	0.893	0.984	0.536
	Delta to PF	−0.211	−0.308	0.081	0.109	−0.068	−0.238	−0.120	−0.248
		0.664	0.566	0.783	0.763	0.893	0.588	0.765	0.653
	Theta to NF	0.318	0.094	0.328	0.334	−0.402	−0.625 *	−0.109	0.329
		0.566	0.793	0.379	0.379	0.295	0.048	0.765	0.536
	Theta to PF	0.047	−0.259	−0.168	−0.132	−0.445	−0.780 **	−0.169	0.108
		0.868	0.584	0.753	0.753	0.240	0.009	0.765	0.765

## Data Availability

The data presented in the current study are available from the corresponding author upon reasonable request. The data are not publicly available due to privacy.
